# Continuous Supply of Non-Combustible Gas Mixture for Safe Autotrophic Culture to Produce Polyhydroxyalkanoate by Hydrogen-Oxidizing Bacteria

**DOI:** 10.3390/bioengineering9100586

**Published:** 2022-10-20

**Authors:** Yuki Miyahara, Chih-Ting Wang, Manami Ishii-Hyakutake, Takeharu Tsuge

**Affiliations:** Department of Materials Science and Engineering, Tokyo Institute of Technology, 4259 Nagatsuta, Midori-ku, Yokohama 226-8502, Japan

**Keywords:** polyhydroxyalkanoate, autotroph, hydrogen-oxidizing bacteria, non-combustible gas, carbon dioxide, *Ralstonia eutropha* (*Cupriavidus necator*)

## Abstract

Polyhydroxyalkanoates (PHAs) are eco-friendly plastics that are thermoplastic and biodegradable in nature. The hydrogen-oxidizing bacterium *Ralstonia eutropha* can biosynthesize poly[(*R*)-3-hydroxybutyrate] [P(3HB)], the most common PHA, from carbon dioxide using hydrogen and oxygen as energy sources. In conventional autotrophic cultivation using *R. eutropha*, a gas mixture containing 75–80 vol% hydrogen is supplied; however, a gas mixture with such a high hydrogen content has a risk of explosion due to gas leakage. In this study, we aimed to develop an efficient cell culture system with a continuous supply of a non-combustible gas mixture (H_2_: O_2_: CO_2_: N_2_ = 3.8: 7.3: 13.0: 75.9) for safe autotrophic culture to produce P(3HB) by hydrogen-oxidizing bacteria, with a controlled hydrogen concentration under a lower explosive limit concentration. When the gas mixture was continuously supplied to the jar fermentor, the cell growth of *R.* *eutropha* H16 significantly improved compared to that in previous studies using flask cultures. Furthermore, an increased gas flow rate and agitation speed enhanced both cell growth and P(3HB) production. Nitrogen source deficiency promoted P(3HB) production, achieving up to 2.94 g/L P(3HB) and 89 wt% P(3HB) content in the cells after 144 h cultivation. *R. eutropha* NCIMB 11599, recombinant *R. eutropha* PHB^-^4, and *Azohydromonas lata* grew in a low-hydrogen-content gas mixture. *R. eutropha* H16 and recombinant *R. eutropha* PHB^-^4 expressing PHA synthase from *Bacillus* *cereus* YB-4 synthesized P(3HB) with a high weight-average molecular weight of 13.5–16.9 × 10^5^. Thus, this autotrophic culture system is highly beneficial for PHA production from carbon dioxide using hydrogen-oxidizing bacteria as the risk of explosion is eliminated.

## 1. Introduction

**Figure 1:**
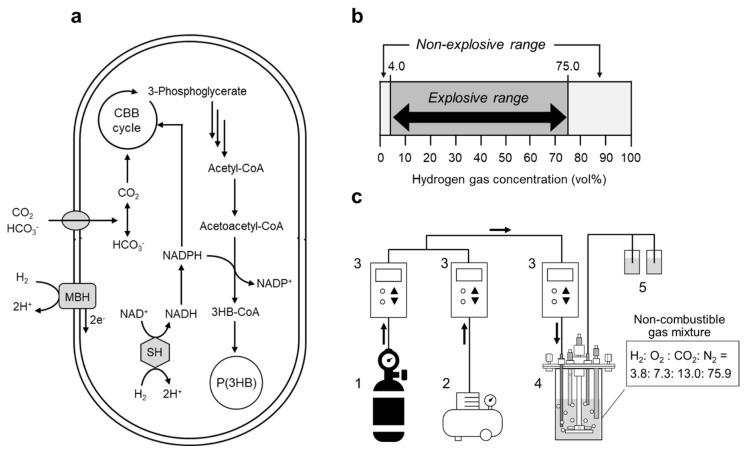
(**a**) Metabolic pathway of P(3HB) biosynthesis from CO_2_ by hydrogen-oxidizing bacterium *R. eutropha*. (**b**) Chart depicting the explosive range for hydrogen gas. (**c**) Experimental setup of the newly developed culture system with a continuous supply of non-combustible gas mixture (H_2_: O_2_: CO_2_: N_2_ = 3.8: 7.3: 13.0: 75.9). The non-combustible gas mixture was continuously supplied by mixing the hydrogen-containing gas mixture (gas cylinder) and air (air compressor) at a volumetric ratio of 2:1 into a 250-mL jar fermentor at 1–15 mL/min. **1**, Hydrogen-containing gas cylinder (H_2_: CO_2_: N_2_ = 5.8: 19.9: 74.3); **2**, air compressor; **3**, mass flow controller; **4**, 250-mL jar fermentor; **5**, 1 N NaOH and 1 N HCl reservoirs for pH adjustment.

Petroleum-based plastics are indispensable in daily life. Thus, the use of plastics has drastically increased, which has negatively affected the environment. According to a recent study, the amount of plastics in oceans will rise to 20–53 million tons per year by the 2030s [1]. Therefore, it is necessary to develop environmentally friendly plastics. Polyhydroxyalknoates (PHAs) are biodegradable polyesters biosynthesized using bacteria from renewable biomass resources, such as sugars, plant oils, and CO_2_. PHAs accumulate in cells as an energy reserve under stress conditions [2]. Thus, PHAs have attracted considerable attention as alternatives to petroleum-based plastics, owing to their biodegradability and sustainability. Generally, PHAs are produced by the bacterial fermentation of plant biomass, which is produced from CO_2_ in the atmosphere through photosynthesis. On the other hand, autotrophic bacteria can directly utilize CO_2_ as a carbon source in PHA production; thus, the total energy consumption required for PHA production can be reduced via direct CO_2_ utilization. This, in turn, can reduce CO_2_ emissions into the atmosphere during plastic production [3]. Based on this idea, the production of PHAs via photosynthesis in cyanobacteria and photosynthetic bacteria has been studied [4]. However, CO_2_ fixation by photosynthetic bacteria requires a large facility for light irradiation and has the disadvantage of a slower growth rate than that of microorganisms used in the fermentation industry [5]. *Ralstonia eutropha* (currently known as *Cupriavidus necator*) is a hydrogen-oxidizing chemoautotrophic bacterium capable of biosynthesizing poly[(*R*)-3-hydroxybutyrate] [P(3HB)], which is the most common PHA, from CO_2_. *R. eutropha* fixes CO_2_ using hydrogen and oxygen as the electron donor and acceptor, respectively (Figure 1a). Furthermore, this bacterium has added advantages for the production of PHAs from CO_2_, owing to its high carbon fixation capacity and high growth rate. In previous studies, *R. eutropha* was autotrophically cultivated by supplying a gas mixture containing a large amount of hydrogen gas (65–85 vol%) [6,7,8,9,10,11,12,13]. However, the explosion gas range of hydrogen is very wide (4–75 vol%), and conventional autotrophic cultivation has the potential risk of explosion due to gas leakage (Figure 1b). This presents a major drawback in autotrophic cultivation, which limits PHA production from CO_2_. Thus, recycled gas closed-circuit culture systems with a lower oxygen partial pressure have been developed to reduce the risk of explosion in a bioreactor [10,12]. Recently, we developed a novel autotrophic culture system using a non-combustible gas mixture with low hydrogen content (less than 4 vol% of H_2_) [14,15]. This system enabled the safe cultivation of *R. eutropha*. However, P(3HB) productivity was very low, with a concentration of 0.27 g/L of P(3HB) obtained after 144 h cultivation [14]. The cellular energy requirement for P(3HB) production is limited, given the low partial pressure of hydrogen gas.

In this study, we established an efficient cell culture system for safe autotrophic culture in order to produce P(3HB) using hydrogen-oxidizing bacteria, which completely eliminated the risk of explosion by continuously supplying a non-combustible gas mixture. Furthermore, we demonstrated the feasibility of PHA production using a low-concentration hydrogen gas mixture.

## 2. Materials and Methods

### 2.1. Strains and Plasmids

The hydrogen-oxidizing bacteria *R. eutropha* H16 (ATCC 17699), *R. eutropha* PHB^-^4 (DSM 541), *R. eutropha* NCIMB 11599, and *Azohydromonas lata* (NBRC 102462^T^, formerly known as *Alcaligenes latus*) were used as host strains for P(3HB) production. A recombinant *R. eutropha* PHB^-^4 strain harboring either the plasmid pBBR1::*phaCAB*_Re_ [16] or pBBR1-*phaRC*_YB4_*AB* [17] was constructed for P(3HB) production. pBBR1::*phaCAB*_Re_ carries the *phaCAB* operon, including the *pha* promoter from *R. eutropha* H16, in the multi-cloning site of pBBR1MCS-2 [18], whereas pBBR1-*phaRC*_YB4_*AB* carries PHA synthase genes from *Bacillus cereus* YB-4 (*phaRC*_YB4_) instead of the *R. eutropha* H16 PHA synthase gene (*phaC*_Re_) in pBBR1::*phaCAB*_Re_. Plasmids were introduced into the cells by electroporation. To yield competent cells for electroporation, the cells were cultured in 100-mL of nutrient-rich medium to an optical density of 0.4–0.6 at a wavelength of 600 nm (OD_600_). The cells were then harvested at 10,000× *g* for 10 min and washed five times with autoclaved cold ultrapure water. The competent cells were suspended in cold water, and approximately 0.5–1.0 μg of desalted plasmid DNA was added. Subsequently, a pulse voltage of 1500 V was applied to the cells in a 2-mm cuvette in order to yield the desired recombinant strains.

### 2.2. Autotrophic Cultivation and P(3HB) Production

Stock cells were grown on NR agar plates (yeast extract, 2 g/L; bonito extract, 10 g/L; tryptone, 10 g/L; agar 2 wt%) at 30 °C overnight. The cells were transferred to 2-mL of NR medium (yeast extract, 2 g/L; bonito extract, 10 g/L; tryptone, 10 g/L) and cultured for 15–20 h for pre-cultivation. Subsequently, 1 vol% of the pre-culture was inoculated into a 250-mL jar fermentor (Bio Jr8, Able Corp., Tokyo, Japan) equipped with mass flow controllers (SEC-E40; CU-2130; S48 32; and MT-51, Horiba, Tokyo, Japan) containing 100-mL of mineral salt (MS) medium (Na_2_HPO_4_ · 12H_2_O, 9 g/L; KH_2_PO_4_, 1.5 g/L; NH_4_Cl, 0.5 g/L; 0.2 g/mL MgSO_4_ · 7H_2_O, 1-mL; and trace element solution, 1-mL) [19]. The composition of the trace element solution was as follows (per liter of 0.1 N HCl): CoCl_2_ · 6H_2_O, 0.218 g; FeCl_3_ · 6H_2_O, 20.5 g; CaCl_2_, 7.8 g; NiCl_2_ · 6H_2_O, 0.118 g; CrCl_3_ · 6H_2_O, 0.105 g; and CuSO_4_ · 5H_2_O, 0.156 g. The culture medium was supplemented with 0.01% (*w*/*v*) antifoam 204 (Sigma-Aldrich, MO, USA) in order to prevent foaming during cultivation. MS media with different concentrations of NH_4_Cl (0.25, 0.50, 1.0, and 1.5 g/L) as a nitrogen source were used to investigate the effects on cell growth and PHA accumulation. When required, kanamycin was added to the medium at a concentration of 100 mg/L for plasmid maintenance. Autotrophic cultivation was performed using a 250-mL jar fermentor with continuous gas flow. A gas mixture of 5.8% H_2_, 19.9% CO_2_, and N_2_ balance (74.3%) supplied from the gas cylinder, and air supplied from the air compressor were mixed at a flow ratio of 2:1 using mass flow controllers. The resultant gas (H_2_: O_2_: CO_2_: N_2_: = 3.8: 7.3: 13.0: 75.9), in which the hydrogen concentration was below the explosion range, was supplied to the jar fermentor at a flow rate of 1–15 mL/min. Typically, 1-mL of culture was collected to measure the OD_600_ and NH_4_Cl concentrations using an ammonia test kit (Fujifilm Wako Pure Chemical, Tokyo, Japan).

### 2.3. Fluorescence Microscopy Analysis

Nile Blue A staining was performed in order to observe P(3HB) granules in the cells. The cultured cells were suspended in 100 μL of ultrapure water and an adequate number of cells were spread on a glass slide. Subsequently, 1 wt% Nile blue A dissolved in 95 vol% ethanol was dropped onto the cells after drying. The samples were then incubated at 55 °C for 10 min. Subsequently, 8 vol% acetic acid solution was dropped, left for 1 min, and then gently washed with water. Cells stained with Nile Blue A were observed under a fluorescence microscope with WU excitation (Olympus, Tokyo, Japan).

### 2.4. Quantification of Intracellular P(3HB)

P(3HB) accumulation in the cells was determined by gas chromatography (GC) analysis using a GC-2014s (Shimadzu, Kyoto, Japan) equipped with an inert cap 1 (GL Sciences, Tokyo, Japan). The freeze-dried cells were methyl esterified with 2-mL of chloroform and 2-mL of methanol: sulfuric acid (85:15) at 100 °C for 140 min [20]. One microliter of the sample was injected, and the heating program was as follows: holding at 90 °C for 2 min, heating to 110 °C at 5 °C/min, and heating to 280 °C at 5 °C/min for 5 min. Intracellular P(3HB) was quantified using the peak area of the 3HB methyl ester and 0.05 vol% methyl octanoate as an internal standard.

### 2.5. Extraction of Polymer and Molecular Weight Analysis

The intracellular P(3HB) polymer was extracted using sodium dodecyl sulfate (SDS) sonication. Approximately 50-mg of the cells was suspended in 4 wt% SDS solution and then ultrasonicated for 10 min at an output level of 12 W. Subsequently, the samples were collected by centrifugation at 10,000× *g* for 10 min and washed at least three times with distilled water before lyophilization for 24 h in order to yield polymer pellets.

The molecular weight of P(3HB) was determined by gel permeation chromatography (GPC). The polymer samples used for the GPC analysis were prepared by dissolving them in chloroform at a concentration of 1 mg/mL and filtering them using a 0.45-μm PVDF membrane. GPC analysis was performed using a Shimadzu Nexera 40 GPC system (Shimadzu, Kyoto, Japan) and a Shodex RI-504 refractive index detector (Showa Denko, Tokyo, Japan) equipped with joint columns of Shodex GPC KF-G 4A and two GPC KF-406LHQ. Chloroform was used as an eluent at a flow rate of 0.3 mL/min. Polystyrene with low polydispersity was used to construct a calibration curve.

## 3. Results

### 3.1. P(3HB) Production by a Continuous Supply of Non-Combustible Gas Mixture

**Figure 2:**
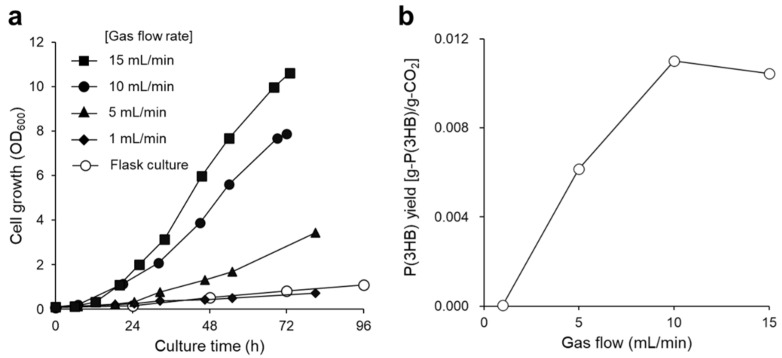
(**a**) Growth curve of *R. eutropha* H16 and (**b**) P(3HB) yield from CO_2_ gas at various gas flow rates. Cells were cultured in MS medium using a safe culture system supplied with the non-combustible gas mixture (H_2_: O_2_: CO_2_: N_2_ = 3.8: 7.3: 13.0: 75.9). The gas mixture was supplied at 1 (filled diamonds), 5 (filled triangles), 10 (filled circles), and 15 mL/min (filled squares), and flask culture [14] was used for comparison (open circles).

Autotrophic cultivation has previously been performed using an Erlenmeyer flask attached to a 1-L aluminum bag containing a non-combustible gas mixture (H_2_: O_2_: CO_2_: N_2_ = 3.6: 7.6: 12.3: 76.5); the gas bag was exchanged every 24 h to avoid the depletion of hydrogen gas [14]. However, cell growth and P(3HB) production were very low due to insufficient gas supply. Thus, to continuously supply the gas mixture and strictly control the gas composition along with the gas flow rate, a 250-mL jar fermentor equipped with a mass flow controller was used for autotrophic cultivation (Figure 1c). First, the effect of the gas flow rate on cell growth and P(3HB) production was explored. Cell growth proportionally increased with the gas flow rate. The highest cell growth was observed at an OD_600_ of 10.6 after 72 h of cultivation with a gas flow of 15 mL/min (Figure 2a). As a result, 69 wt% P(3HB) in the cells and 1.95 g/L P(3HB) were obtained using a jar fermentor with a continuous supply of a non-combustible gas mixture.

**Figure 3:**
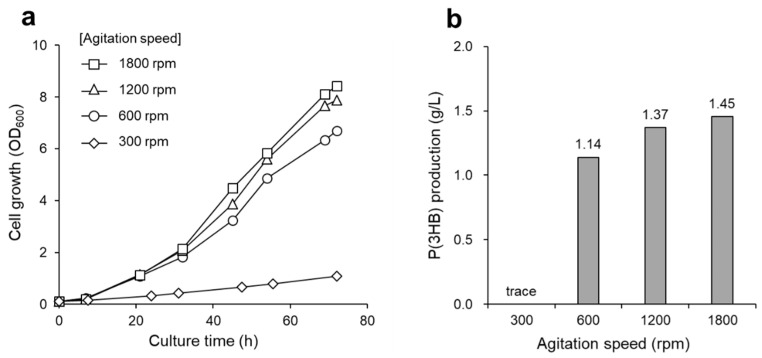
(**a**) Growth curve and (**b**) P(3HB) production of *R. eutropha* H16 at various agitation speeds. Cells were cultured at 30 °C for 72 h and a non-combustible gas mixture was supplied continuously at 10 mL/min. Agitations speeds were as follows: 300 (diamonds), 600 (circles), 1200 (triangles), and 1800 rpm (squares).

The highest P(3HB) yield from the supplied CO_2_ gas was 0.011 g-P(3HB)/g-CO_2_ [carbon capture was 0.023 g-carbon of P(3HB)/g-carbon of CO_2_], and the polymer yield peaked at a gas flow rate of 10 mL/min, clearly indicating that this condition effectively produced P(3HB) from CO_2_ (Figure 2b). Therefore, a gas flow rate of 10 mL/min was used in the subsequent experiments. Subsequently, the agitation speed was optimized to adequately dissolve the gas mixture as a substrate in the culture medium. It is known that a high agitation speed is beneficial for enhancing cell growth and P(3HB) production (Figure 3). However, no differences in P(3HB) production were observed at agitation speeds of 1200 and 1800 rpm. This result suggests that dissolution of the substrate gas becomes saturated in the culture medium at over 1200 rpm. The P(3HB) yield from the supplied hydrogen under optimal culture conditions was 0.832 [g-P(3HB)/g-H_2_]. It has been reported that 33 mol of hydrogen is required for the autotrophic synthesis and polymerization of 1 mol of 3HB units from a high-hydrogen-content gas [6,12]. Assuming that this stoichiometry does not vary under the conditions with a low-hydrogen-content gas, P(3HB) production in this study reached 64% of the theoretical yield.

### 3.2. Effect of Nitrogen Source on P(3HB) Accumulation

**Figure 4:**
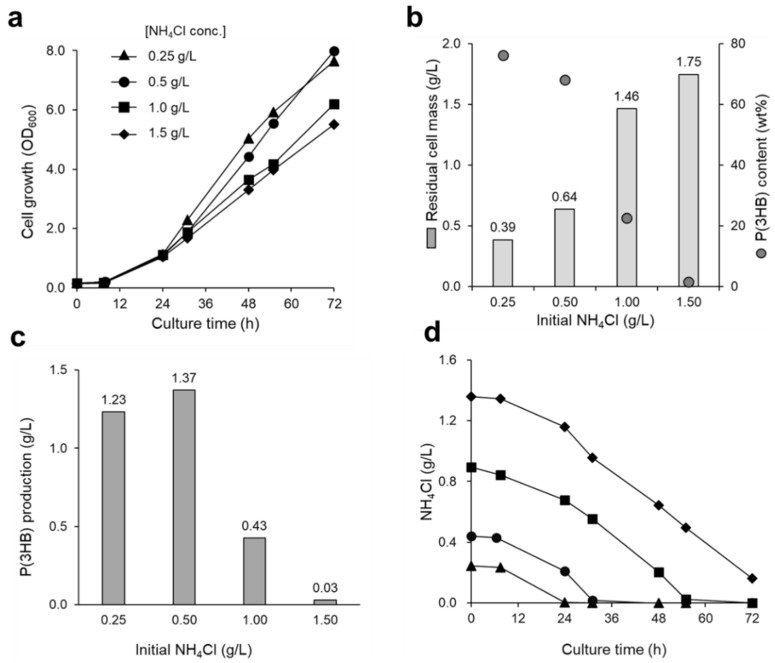
The effect of nitrogen source concentration in culture medium on (**a**) cell growth and (**b**,**c**) P(3HB) accumulation of *R. eutropha* H16. The cells were cultured in MS medium containing low (0.25 and 0.5 g/L) and high concentrations (1.0 and 1.5 g/L) of NH_4_Cl, with the gas mixture supplied at 10 mL/min. (**d**) The changes in nitrogen source (NH_4_Cl) concentration in the culture medium were monitored. The initial NH_4_Cl concentrations were 0.25 (triangles), 0.5 (circles), 1.0 (squares), and 1.5 g/L (diamonds).

Nitrogen source is a nutrient that is closely related to cell growth and P(3HB) accumulation [14,21]. To obtain favorable cell growth and P(3HB) accumulation, it is necessary to investigate the effects of nitrogen source in the culture medium. Therefore, the effects of NH_4_Cl, which was used as a nitrogen source supplementing the mineral medium at various concentrations (0.25, 0.5, 1.0, and 1.5 g/L), on cell growth and P(3HB) accumulation were investigated. Favorable cell growth was observed in both low (0.25 and 0.5 g/L) and high (1.0 and 1.5 g/L) concentrations of NH_4_Cl-containing medium (Figure 4a). In a medium with a low concentration of nitrogen source, a high amount of P(3HB) accumulated in the cells. As for the initial NH_4_Cl concentrations of 0.25 g/L and 0.5 g/L, 76 wt% and 68 wt% of P(3HB) accumulated, respectively (Figure 4b). However, P(3HB) accumulation decreased with increasing NH_4_Cl concentration, and P(3HB) hardly accumulated in the medium with 1.5 g/L NH_4_Cl.

**Figure 5:**
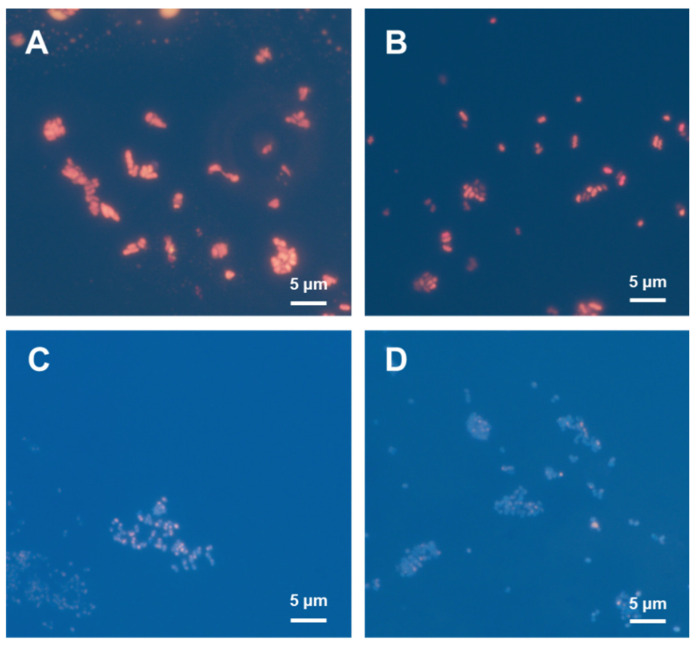
Nile Blue A staining assays of *R. eutropha* H16 cultured in MS medium at different NH_4_Cl concentrations of (**A**) 0.25, (**B**) 0.5, (**C**) 1.0, and (**D**) 1.5 g/L under fluorescence microscopy.

Intracellular P(3HB) granules stained with Nile Blue A emit orange fluorescence under UV excitation [22]. Nile Blue A staining was applied to the cells in order to observe the accumulation of P(3HB) granules under different NH_4_Cl concentrations (Figure 5). The cells cultured using a low NH_4_Cl concentration showed strong fluorescence intensity because of a high amount of P(3HB) accumulation. In contrast, fluorescence intensity was weaker for the cells cultured in a medium with a high NH_4_Cl concentration. The results from the fluorescence microscopy analysis correlated with the GC results for P(3HB) quantification, as expected; thus, it is evident that nitrogen source limitation promotes P(3HB) accumulation (Figure 4b and Figure 5). Moreover, the shape of the cells obtained from low and high NH_4_Cl concentrations were different. Under limited nitrogen source conditions, the cells were enlarged and appeared to be oblong, suggesting high P(3HB) accumulation. Meanwhile, the cells grown in medium containing high nitrogen source concentrations were small and round, corresponding to low P(3HB) accumulation.

Depletion of NH_4_Cl in the low-nitrogen-source medium was observed from 24 to 48 h and resulted in a drastic increase in OD_600_ (Figure 4a,d). Conversely, in the media supplemented with high concentrations of NH_4_Cl, 0.2–0.6 g/L NH_4_Cl remained after 48 h of cultivation. Interestingly, both the desired cell growth and high P(3HB) accumulation were achieved in the culture medium supplemented with 0.5 g/L of NH_4_Cl, in which 1.37 g/L of P(3HB) was obtained after 72 h of cultivation (Figure 4c). Furthermore, by extending the culture time to 144 h, P(3HB) production and P(3HB) content in the cells increased to 2.94 g/L and 89 wt%, respectively (Table 1). Hence, limited nitrogen source conditions are essential during the P(3HB) accumulation phase in order to maximize polymer production.

### 3.3. Hydrogen-Oxidizing Bacteria Grown in a Safe Culture System with a Low-Hydrogen-Content Gas Mixture

**Figure 6:**
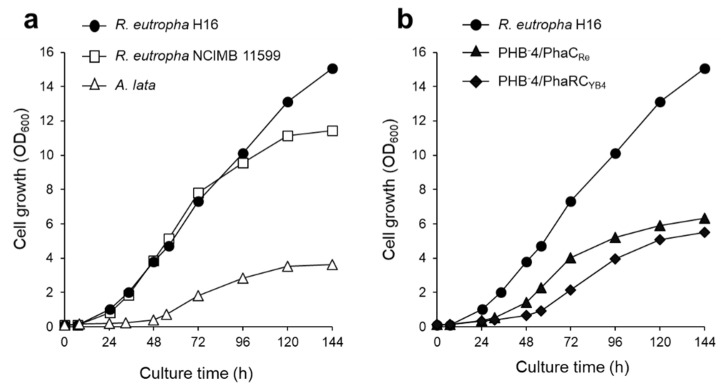
(**a**) Growth curve of different bacteria, *R. eutropha* H16 (closed circle), *R. eutropha* NCIMB 11599 (open squares), and *A. lata* (open triangles). (**b**) Growth curve of *R. eutropha* H16 (closed circle), PHB^-^4/PhaC_Re_ (closed triangles), and PHB^-^4/PhaRC_YB4_ (closed diamonds). All strains were cultured by supplying a low-hydrogen-content gas in MS medium at 10 mL/min with an agitation speed of 1200 rpm.

*R. eutropha* NCIMB 11599 [23,24,25,26] and *Azohydromonas lata* [27,28] are hydrogen-oxidizing bacteria with high P(3HB) production capacity. Therefore, we applied these bacteria to a culture system with low hydrogen gas supply. The bacterial strains were cultured by continuously supplying gas at a flow rate of 10 mL/min. The dry cell weight and P(3HB) production of *R. eutropha* NCIMB 11599 were comparable to those of *R. eutropha* H16, with 2.46 g/L of dry cell weight and 1.79 g/L of P(3HB) production, respectively (Table 1). In contrast, the growth of *A. lata* was moderate compared to that of *R. eutropha*, with an OD_600_ of 3.6 after 144 h of cultivation (Figure 6a). Furthermore, the P(3HB) content of *A. lata* was less than 27 wt%. The inefficiency of P(3HB) accumulation in *A. lata* can be attributed to its inherent hydrogenase activity. The usual hydrogenase is highly oxygen-sensitive; thus, it is likely that a continuous supply of oxygen (7.3 vol%) might have suppressed hydrogenase activity during autotrophic cultivation [29]. Hence, it is necessary to optimize cultivation conditions when *A. lata* is used for P(3HB) production via autotrophic cultivation.

### 3.4. Autotrophic P(3HB) Production by Using Recombinant Strains

The material properties of PHAs are dependent on their molecular weight and monomer composition, which are controlled by PHA synthases. Various PHA synthases have been used for the production of PHAs in the heterotrophic cultivation of *R. eutropha* [19,30,31,32,33,34,35,36]. PhaRC_YB4_ derived from *B. cereus* YB-4 produces high-molecular-weight P(3HB) from fructose, gluconate, and plant oils as the sole carbon source [17]. The feasibility of culturing recombinant strains with a low-hydrogen-content gas was also investigated. For this purpose, autotrophic P(3HB) production was performed using PhaRC_YB4_- or PhaC_Re_ (derived from *R. eutropha* H16)-expressing recombinant strains. Figure 6b shows that the growth rate of the recombinant strain was lower than that of the wild-type strain (H16 strain). It is possible that the high energy required by the cells to retain and overexpress the PHA synthase plasmid may have hindered their growth. P(3HB) accumulation of 67 wt% and 59 wt% was achieved in PHB^-^4/PhaC_Re_ (PhaC_Re_-expressing strain) and PHB^-^4/PhaRC_YB4_ (PhaRC_YB4_-expressing strain), respectively (Table 1). Furthermore, the PHA produced by the recombinant strains was confirmed to be a P(3HB) homopolymer by ^1^H NMR analysis (data not shown). In addition, PHB^-^4/PhaRC_YB4_ produced P(3HB) with a high weight-average molecular weight (*M_w_*) of 16.9 × 10^5^, which was similar to the H16 strain. Collectively, these results demonstrate the feasibility of safe autotrophic culture using a non-combustible gas mixture for recombinant strains.

## 4. Discussion

The biosynthesis of P(3HB) via autotrophic cultivation by hydrogen-oxidizing bacteria requires a large supply of hydrogen in the bioreactor. A new culture strategy employing a low-oxygen-content gas, which has a partial pressure of less than 6.9 vol%, has been developed to reduce the risk of explosion from hydrogen gas [7,12]. Under culture conditions with a low partial pressure of oxygen, it is necessary to use a bioreactor with a high overall oxygen transfer coefficient (*K*_L_*a*) in order to obtain sufficient cell growth. To achieve this, a basket-type agitation system [12] and air-lift bioreactor [8] were used. Two-stage cultivation, combined with autotrophic and heterotrophic cultivation, has also been implemented [9,10,11,37]. These studies succeeded in reducing the risk of explosion. Nonetheless, the risk of explosion still exists due to the potential for gas leakage. Recently, we developed a closed, low-hydrogen system in which a 500-mL flask was filled with 3.6 vol% hydrogen for autotrophic cultivation in order to completely eliminate the risk of explosion [14]. Although it was a safer culture system, the resultant cell growth and P(3HB) yield were unsatisfactory, owing to insufficient hydrogen supply. Therefore, in this study, we established a new and safe autotrophic culture system to improve cell growth and P(3HB) production from a non-combustible gas mixture. By continuously supplying the gas mixture to the jar fermentor, the cell growth of *R. eutropha* H16 was significantly improved compared with previous studies using flask cultures [14]. Additionally, cell growth and P(3HB) production using the culture system developed in this study were improved by increasing the gas flow rate and agitation speed (Figure 2 and Figure 3); up to 1.95 g/L of P(3HB) was produced after 72 h of cultivation. This indicates that the demand for hydrogen gas required for cell growth and P(3HB) accumulation was fulfilled through the continuous supply of gas with a low hydrogen content. Furthermore, a high agitation speed promoted the dissolution of the gas mixture into the culture medium. Notably, improved cell growth and P(3HB) accumulation were achieved, even below the lower explosive limit of hydrogen.

*R. eutropha* H16 accumulates P(3HB) for energy storage inside cells when nutrient sources, such as nitrogen and phosphate which are required for cell growth, are deficient [10,14,38,39]. In this study, high amounts of P(3HB) accumulated in the cells using a culture medium with a low nitrogen source concentration, consistent with a previous study [14] (Figure 4). In addition, under low nitrogen source concentration conditions, nitrogen source depletion occurred after 24–48 h of cultivation and increased the OD_600_ drastically, corresponding to enlarged cell size (Figure 4 and Figure 5). These results suggest that nitrogen source deficiency in cells may trigger P(3HB) accumulation. By optimizing the gas flow rate, agitation speed, and nitrogen source concentration, P(3HB) production and accumulation levels were increased up to 2.94 g/L and 89 wt%, respectively (Table 1). The P(3HB) yield was 10.9-fold higher than that reported in a previous study [14]. Therefore, efficient P(3HB) production can be achieved by switching to nitrogen-source-limited conditions after obtaining sufficient cell growth with a gas mixture that is continuously supplied, even when using a non-combustible gas mixture.

PHA synthases (PhaCs) not only catalyze the polymerization reaction, but also affect the monomer composition and molecular weight of PHA, which in turn determines the material properties of the resultant PHA. PhaCs derived from *Aeromonas caviae* and *Pseudomonas* sp. 61-3 have wide substrate specificities. Therefore, these PhaCs were evaluated in recombinant *R. eutropha* to produce PHA copolymers incorporating 3-hydroxyhexanoate (3HHx) and/or 3-hydroxy-4-methylvalerate (3H4MV) monomers into 3HB polymer chains, which are much more practical PHAs owing to their better flexibility [14,19,30,31,32,33,34,40]. In addition, ultrahigh-molecular-weight P(3HB) [UHMW-P(3HB)], with a molecular weight of several millions, shows excellent mechanical properties and high processability [41,42]. Recombinant *R. eutropha* expressing PhaRC_YB4_ produces high amounts of P(3HB) with a relatively high molecular weight (*M_w_* up to 400 × 10^5^) from sugars and plant oils [17]. The recombinant strain was capable of synthesizing P(3HB) with a high molecular weight (*M_w_* = 16.9 × 10^5^) even under autotrophic culture with the supply of a non-combustible gas mixture (Table 1). This finding suggests that the developed culture system is beneficial for PHA production using not only *R. eutropha* H16 (wild-type), but also recombinant strains. Therefore, efficient autotrophic cultivation for PHA production using metabolically engineered *R. eutropha* strains is expected to produce PHA copolymers and/or UHMW-P(3HB).

## 5. Conclusions

In this study, we established a new and safe autotrophic culture system that completely eliminated the explosion risk from hydrogen gas, while efficiently culturing cells by continuously supplying a non-combustible gas mixture (H_2_: O_2_: CO_2_: N_2_ = 3.8: 7.3: 13.0: 75.9). Both cell growth and P(3HB) production were improved with an increased gas flow rate and agitation speed. Additionally, nitrogen source deficiency in the medium promoted P(3HB) production, achieving up to 2.94 g/L P(3HB) and 89 wt% P(3HB) accumulation in the cells. The P(3HB) yield was 10.9-fold higher than that reported in a previous study [14]. Furthermore, the efficient cell culture system developed in this study is beneficial for autotrophic cultures using various hydrogen-oxidizing bacteria and recombinant strains. These results encourage the future development of practical PHAs production from CO_2_ using hydrogen-oxidizing bacteria.

## Figures and Tables

**Table 1:** P(3HB) production by various strains grown in MS medium with a low-hydrogen-content gas mixture supply (10 mL/min).

Strain	Dry Cell wt. (g/L)	Residual Cell Mass (g/L)	PHA Content (wt%)	PHA (g/L)	Molecular Weight ^a^	
					*M_w_* (×10^5^)	PDI
*R. eutropha* H16	3.31	0.37	89	2.94	13.5	1.87
*R. eutropha* NCIMB 11599	2.46	0.67	73	1.79	-	-
*A. lata*	0.83	0.61	27	0.22	-	-
*R. eutropha* PHB^-^4/PhaC_Re_	1.55	0.51	67	1.04	1.54	1.39
*R. eutropha* PHB^-^4/PhaRC_YB4_	1.17	0.48	59	0.69	16.9	1.57
*R. eutropha* H16/pBBR1MCS-2 ^b^ (Flask culture)	0.39	0.12	70	0.27	-	-

Cells were cultured in a 250-mL jar fermentor with a low-hydrogen-content gas mixture (3.8% H_2_, 7.3% O_2_, 13.0% CO_2_, 75.9% N_2_) at 10 mL/min and 30 °C for 144 h. ^a^ Molecular weight was determined by GPC analysis. *M_w_*, weight-average molecular weight; PDI, polydispersity index. ^b^ *R. eutropha* H16/pBBR1MCS-2 (empty plasmid) was cultured for 144 h in Erlenmeyer flasks attached to a 1-L aluminum bag containing a non-combustible gas mixture, and the gas bag was exchanged every 24 h [14].

## Data Availability

Not applicable.

## Abbreviations

Polyhydroxyalkanoates, PHAs; poly[(*R*)-3-hydroxybutyrate], P(3HB); weight-average molecular weight, *M_w_*; PHA synthases, PhaCs; *R. eutropha* H16 PHA synthase, PhaC_Re_; *Bacillus cereus* YB-4 PHA synthase, PhaRC_YB4_; nutrient rich medium, NR medium; mineral salt medium, MS medium; gas chromatography, GC; gel permeation chromatography, GPC; 3-hydroxyhexanoate, 3HHx; 3-hydroxy-4-methylvalerate, 3H4MV; ultrahigh-molecular-weight P(3HB), UHMW-P(3HB).
